# High-frequency electrical stimulation attenuates neuronal release of inflammatory mediators and ameliorates neuropathic pain

**DOI:** 10.1186/s42234-022-00098-8

**Published:** 2022-10-05

**Authors:** Huan Yang, Timir Datta-Chaudhuri, Sam J. George, Bilal Haider, Jason Wong, Tyler D. Hepler, Ulf Andersson, Michael Brines, Kevin J. Tracey, Sangeeta S. Chavan

**Affiliations:** 1 Institute for Bioelectronic Medicine, Feinstein Institutes for Medical Research, 350 Community Drive, Manhasset, NY 11030 USA; 2 Elmezzi Graduate School of Molecular Medicine, Feinstein Institutes for Medical Research, Northwell Health, Manhasset, NY USA; 3 Donald and Barbara Zucker School of Medicine at Hofstra/Northwell, Hempstead, NY USA; 4 Department of Women’s and Children’s Health, Karolinska Institute, Karolinska University Hospital, 17176 Stockholm, Sweden

**Keywords:** Pain, Inflammation, Nerve stimulation, HMGB1, CGRP, Substance P, Nerve injury, Mechanical hyperalgesia, Allodynia

## Abstract

**Background:**

Neuroinflammation is an important driver of acute and chronic pain states. Therefore, targeting molecular mediators of neuroinflammation may present an opportunity for developing novel pain therapies. In preclinical models of neuroinflammatory pain, calcitonin gene-related peptide (CGRP), substance P and high mobility group box 1 protein (HMGB1) are molecules synthesized and released by sensory neurons which activate inflammation and pain. High-frequency electrical nerve stimulation (HFES) has achieved clinical success as an analgesic modality, but the underlying mechanism is unknown. Here, we reasoned that HFES inhibits neuroinflammatory mediator release by sensory neurons to reduce pain.

**Methods:**

Utilizing in vitro and in vivo assays, we assessed the modulating effects of HFES on neuroinflammatory mediator release by activated sensory neurons. Dorsal root ganglia (DRG) neurons harvested from wildtype or transgenic mice expressing channelrhodopsin-2 (ChR2) were cultured on micro-electrode arrays, and effect of HFES on optogenetic- or capsaicin-induced neuroinflammatory mediator release was determined. Additionally, the effects of HFES on local neuroinflammatory mediator release and hyperalgesia was assessed in vivo using optogenetic paw stimulation and the neuropathic pain model of chronic constriction injury (CCI) of the sciatic nerve.

**Results:**

Light- or capsaicin-evoked neuroinflammatory mediator release from cultured transgenic DRG sensory neurons was significantly reduced by concurrent HFES (10 kHz). In agreement with these findings, elevated levels of neuroinflammatory mediators were detected in the affected paw following optogenetic stimulation or CCI and were significantly attenuated using HFES (20.6 kHz for 10 min) delivered once daily for 3 days.

**Conclusion:**

These studies reveal a previously unidentified mechanism for the pain-modulating effect of HFES in the setting of acute and chronic nerve injury. The results support the mechanistic insight that HFES may reset sensory neurons into a less pro-inflammatory state via inhibiting the release of neuroinflammatory mediators resulting in reduced inflammation and pain.

**Supplementary Information:**

The online version contains supplementary material available at 10.1186/s42234-022-00098-8.

## Introduction

Pain is a critical defensive mechanism which serves to protect the body from injury and promote healing of damaged tissues. While acute pain serves to avoid further injury, chronic pain is a major clinical challenge that, if unmet, significantly diminishes quality of life in the affected individuals. Chronic pain affects around 20-50 million US adults (Dahlhamer et al. 2018; Nahin 2015), and 1.5 billion people worldwide (Gureje et al. 1998; Elzahaf et al. 2012). It limits daily activities (Gureje et al. 1998; Smith et al. 2001), and is associated with reduced work productivity (Stewart et al. 2003; Yelin 2003), anxiety and depression (Turk et al. 2010), suicidality (Petrosky et al. 2018) and overall reduced quality of life (Stewart et al. 2003; Yelin et al. 2007). The economic burden associated with chronic pain is significant, with an annual cost exceeding $600 Billion in the US (Committee on Advancing Pain Research, 2011) (Gaskin and Richard 2012). Opioids have been widely used for the management of chronic pain. However, an overreliance on the prescription of opioids for chronic pain has resulted in a public health crisis with high levels of addiction and death (Ballantyne and Mao 2003; Martell et al. 2007). Other therapies, e.g., non-steroidal anti-inflammatory drugs, often have limited efficacy and have risks of potential serious adverse effects. Clearly, novel solutions for developing alternative pain treatment options are urgently needed.

Pain is initiated and maintained by activation of sensory neurons (nociceptors) which innervate the muscles, visceral tissues, bones, gastrointestinal tract, genitourinary tract, cornea and skin (Gold and Gebhart 2010). Nociceptors not only detect changes in the body’s internal and external milieu and relay the information to the nervous system, but also send antidromic signals back into the target tissues and activate inflammation (neuroinflammation) leading to pain and tissue injury (Chavan et al. 2017; Bennett and Xie 1988; Yang et al. 2021a; Cohen et al. 2019). Multiple molecules synthesized and released by nociceptors have been identified as important neuroinflammatory mediators. These include calcitonin gene related peptide (CGRP) and substance P, neuropeptides which have been implicated in the development of pain states and targeted for analgesic therapy (Massaad et al. 2004). Recently, high mobility group box 1 (HMGB1) has been identified as a key pro-inflammatory protein released into the local microenvironment by activated nociceptors following nerve injury (Yang et al. 2021a, b). Ablation of neuronal HMGB1 or neutralization of extracellular HMGB1 protects from neuroinflammation and hyperalgesia in preclinical models of nerve injury indicating that nociceptor HMGB1 is an upstream mediator of neuroinflammation (Yang et al. 2021a). Although the role of multiple neuroinflammatory mediators is reasonably well defined in the generation of neuroinflammation and pain, considerably less is known about the strategies to regulate their release by activated nociceptors.

High-frequency electrical stimulation (HFES) in the kilohertz frequency range is an evolving therapy for chronic pain management (Kumar et al. 2007; Echeverria-Villalobos et al. 2021; Youn et al. 2015) with limited side effects compared to chronic pharmacological treatments (Kumar et al. 2007; Chakravarthy et al. 2019; Tieppo Francio et al. 2021). Clinically, it has been used to stimulate both the spinal cord and the dorsal root ganglia (Arle et al. 2016; Annemans et al. 2014; Luecke et al. 2020; Al-Kaisy et al. 2014) for multiple indications including sciatica (Dewberry et al. 2021), failed back surgery syndrome (Reverberi et al. 2013; Frey et al. 2009), complex regional pain syndrome (Turner et al. 2004; Kemler et al. 2008) and diabetic polyneuropathy (Heijmans and Joosten 2020; Pluijms et al. 2012), while avoiding paresthesia. Since its development in the 1960s as an implantable spinal cord stimulation system, the technology has evolved substantially into non-invasive stimulation devices. One such advancement is the TrueRelief® device which received Food and Drug Administration clearance in 2021. This transdermal electrical nerve stimulator is used by medical professionals to deliver high-frequency electrical current transcutaneously as a therapy for acute or chronic pain. Despite growing interest in and extensive clinical application of high-frequency stimulation, the molecular mechanisms underlying the observed therapeutic efficacy remains poorly characterized. Considering the crucial role of neuroinflammatory mediators in nociceptor mediated pain states, we reasoned that HFES may inhibit their release. Here, utilizing optogenetic, pharmacologic, and injury-related activation of nociceptors in vitro and in vivo, we show that HFES reduces nociceptor neuroinflammatory mediator release and significantly attenuates hyperalgesia.

## Materials and methods

### Animals

All procedures with experimental animals were approved by the Institutional Animal Care and Use Committee and the Institutional Biosafety Committee of the Feinstein Institute for Medical Research, Northwell Health, Manhasset, NY in accordance with NIH guidelines and the ethical guidelines of the International Association for the Study of Pain. Animals were maintained at 25 °C on a 12-h light-dark cycle with free access to food and water. Sprague-Dawley rats, C57BL/6 mice, VGlut2-ires-Cre (*Slc17a6*^*tm2(cre)Lowl*^/J), and ChR2-YFP (yellow fluorescent protein)-flox mice (B6.Cg-*Gt(ROSA) 26Sor*^tm32(CAG-COP4*H134R/EYFP)Hze^/J) were obtained from Jackson Laboratories (Bar Harbor, ME). Animals were acclimated for 7 days before any experimental use, and housed under standard temperature, light and dark cycles. Mice (8 to 12 weeks old) and rats (2 to 3 months old) were used in these studies. VGlut2-ires-Cre mice were bred with ChR2-YFP-flox mice to generate Vglut2-Cre/ChR2-YFP mice which express ChR2 allele under control of the *Vglut2* locus, which encodes vesicular glutamate transporter type 2 which is predominantly expressed in peripheral sensory neurons. The genotypes of the transgenic strains were confirmed using PCR (Transnetyx, Cordova, TN).

### Neuronal cultures

The L_1_ to L_6_ dorsal root ganglia (DRG) from adult Vglut2-Cre/ChR2-YFP mice (8–12 weeks old) were dissected and dissociated in collagenase/dispase II (1 mg/ml, Roche Applied Science, Indianapolis, IN) in Hanks’ balanced salt solution (HBSS) at 37 °C for 90 min. The DRGs were triturated and cells filtered using 70 μm nylon cell strainer with centrifugation. After centrifugation, cell pellets were suspended in neurobasal-A medium (ThermoFisher Scientific, Waltham, MA), supplemented with neural growth factor (50 ng/ml, ThermoFisher Scientific), 1X B27 supplement (ThermoFisher Scientific), penicillin (ThermoFisher Scientific), and streptomycin (Thermo Fisher Scientific). The cells were then plated on coverslips pre-coated with poly-L lysine (100 μg/ml, Sigma-Aldrich, St. Louis, MO) and laminin (50 μg/ml, Sigma-Aldrich), and allowed to adhere for 12 to 15 h at 37 °C (with 5% CO_2_) and used at 48–72 h following plating.

### Stimulation of DRG neurons with capsaicin

Primary DRG neurons were isolated from C57BL/6 mice and cultured in poly-lysine (100 μg/ml) and laminin (50 μg/ml) coated chamber plates for 48 h. Cells were stimulated with capsaicin (5 μM, Sigma-Aldrich) with or without simultaneous HFES. Cell supernatant was collected at 60 min for further analysis.

### Optogenetic stimulation of DRG neurons

Primary DRG neurons isolated from Vglut2-Cre/ChR2-YFP and TRPV1-Cre/ChR2-YFP mice were cultured on poly-lysine (100 μg/ml) and laminin (50 μg/ml) coated chamber plates for 48 h. Cultured cells were activated with light stimulation using 470 nm or control yellow at 595 nm light delivered by a light emitting diode at 20 Hz, 10% duty cycle for 60 min (DCZ100, ThorLabs, Newton, New Jersey), with or without simultaneous HFES. The cell supernatant was collected at the end of 60 min of light stimulation.

### High-frequency electrical stimulation using multi-electrode array (MEA) plates

Prior to plating, 60 PEDOT-CNT and TiN electrodes MEA plates (30 μm diameter, 200 μm spacing, Multichannel Systems, Reutlingen, Germany) were sterilized by soaking with 75% alcohol for 30 min and coated with poly-L lysine (100 μg/ml) and laminin (50 μg/ml) at 4 °C overnight. DRG neurons were isolated from Vglut2-ChR2-YFP or C57BL/6 mice as described and plated at approximately 5000-10,000 cells/well. Cells were cultured for 48-72 h at 37 °C (with 5% CO_2_) before stimulation. Cells were stimulated with 470 nm LED light at 20 Hz, 10% duty cycle for 30 min (Daou et al. 2013). Concurrently, HFES (10 kHz, 2 mA, rectangular symmetric, biphasic, charge balanced, Multichannel Systems STG4008) was performed for 30 min by assigning half of the electrodes (opposing quadrants were grouped together) to each of the two stimulation terminals. Electrical stimulation was initiated a few seconds before optical stimulation, and optical stimulation was turned off a few seconds before turning off the electrical stimulation, to ensure that the neurons did not receive optical stimulation without receiving simultaneous HFES. After an additional 60 min, cell supernatants were collected for analyses. After each experiment, MEA plates were cleaned for reuse by soaking with an enzymatic cleaner (1% Tergazyme in PBS, Alconox Inc., White Plains, NY) overnight to ascertain the complete removal of cell debris. Between uses the MEAs were filled with sterile distilled water and stored at 4 °C in the dark to prevent microbiological contaminations and to maintain a hydrophilic surface.

### Chronic constriction injury (CCI) of the sciatic nerve

CCI of the sciatic nerve was performed as described previously (Yang et al. 2021a). Briefly, mice and rats were anesthetized with isoflurane inhalation (induction level of 4 and 2% maintenance) and the surgical field was shaved. Under anesthesia and aseptic surgical conditions, the sciatic nerve was gently isolated by separating the biceps femoris and gluteus superficialis, and loosely ligated with 5/0 Ethicon chromic catgut suture (3 sutures for mice and 4 sutures for rats). In sham-operated animals, the sciatic nerve was exposed, but not ligated. The muscle was then closed with 4–0 silk sutures (5-0 vicryl violet), while skin incision was closed using skin clips. Following surgery, the animals were allowed to recover for 2 weeks before any assessment.

### High-frequency electrical stimulation

HFES of animals was performed using the TrueRelief® device (TrueRelief, Santa Monica, CA), which has received approval of the US FDA as a pain-modulating device for the treatment of acute and chronic pain. Current, at a ultra high-frequency of 20.6 kHz, is delivered via two stainless steel probes which are placed in direct contact with the skin at the target site. Two weeks post-CCI surgery, animals were anesthetized with isoflurane (1.5-2%) and stimulated transcutaneously using the TrueRelief® device. Conductive gel (Spectra 360 Electrode Gel) was applied to the tips of the two electrical probes, which were then placed on the skin at the site of CCI surgery (approximately 1 cm apart for mice and 2 cm apart for rats). High-frequency electrical stimulation (20.6 kHz) was applied to the surgical incision site for a total of 10 min as follows: across the incision, with one probe medial to, and one probe lateral to the incision for 5 min; and along the incision, with one probe just outside one end of the incision and the other just outside the other end of the incision, for 5 min. This process was performed once a day for either 1 or 3 days. Control groups (sham stimulation groups) received only anesthesia. Mechanical hypersensitvity was assessed 24 h post-treatment using calibrated von Frey filaments.

### Simultaneous optogenetic and high frequency stimulation

Vglut2-ChR2-YFP or C57BL/6 mice were anesthetized with isoflurane (1.5-2%), and stimulated directly on the right hind paw at 470 nm (4.7 mW, 3 Hz, 20% duty cycle) for 15 min (Supplement Fig. 2). In control experiments, 595 nm stimulation was used (Daou et al. 2013). Immediately after optogenetic stimulation, the animals received HFES (20.6 kHz) using the two probes directly positioned dorsal to the right sciatic nerve approximately 1 cm apart and applied parallel and perpendicular to the sciatic nerve for 5 min in each direction (total stimulation time of 10 min). Mechanical hypersensitivity was measured after 5 h. In some experiments, animals were euthanized and the right hind paw was collected for biochemical analysis.

### EXPEL method

To extrude the interstitial fluid from paw tissue, an innovative methodology named EXPEL was adapted as previously described (Costanza et al. 2018). Fresh paw tissue (about 0.5 g) was collected immediately after harvesting, cut into 3 mm pieces, and placed in a 10 ml syringe. Four hundred microliter of hypertonic extraction buffer [500 ml PBS supplemented with 4.5 g NaCl and 2X protease inhibitor (Sigma-Aldrich)] was added to the tissue. The plunger was then set to 5 ml line (allowing an intake of approximately 4 ml air-bubble), followed by alternating pressure for 1 min, by moving the plunger from 5 ml to 1 ml line, repeating this procedure for a total of 30 times for each sample. The EXPEL-extruded fluid was collected and stored at − 20 °C for further analysis.

### Measurements of HMGB1, calcitonin gene-related peptide (CGRP), substance P, and LDH

Levels of HMGB1 in the paw tissue or cell supernatants were quantitated using ELISA kit (IBL International, Hamburg, Germany); CGRP was measured using EIA kits (Cayman Chemical Company, Ann Arbor, MI) and substance P via EIA kits (R&D Systems, Minneapolis, MN). Media LDH content was also determined using a detection kit (Cayman Chemical Company, Ann Arbor, MI). Total protein content was measured using Bradford assay (BioRad, Hercules, CA).

### Mechanical hypersensitivity analysis

Mechanical hypersensitivity was assessed using von Frey filaments and the Dixon up-down method. Animals were allowed to acclimatize in the testing apparatus on a metal mesh floor for 30 min before testing. For assessment, the animal was placed on an elevated mesh platform, and filaments (von Frey 1894) (exerting forces of 0.4-7.3 g, Ugo Basile, Varese, Italy) were inserted through the mesh to stimulate the plantar aspect of the hindpaw in ascending order to define the threshold stimulus intensity required to elicit a paw withdrawal response. The filament was held in place for stimulation for approximately 5–7 s and repeated for each paw after an interval of at least 5 min. The behavioral responses were then used to calculate absolute threshold (50% probability of response) as described previously (Milligan et al. 2000).

### Statistical analysis

Data were analyzed using Graphpad Prism software. Data are presented as means ± SEM unless otherwise stated. Differences between treatment groups were determined by Student’s t test, one-way ANOVA, and two-way ANOVA followed by Tukey’s multiple comparisons multiple comparison tests. *P* values less than 0.05 were considered statistically significant.

## Results

### HFES attenuates neuroinflammatory mediator release by activated sensory neurons

We and others have previously demonstrated that activated sensory neurons release HMGB1, substance P and CGRP (Yang et al. 2021a; Seybold 2009; Lukacs et al. 2017), however, whether there are differences in the temporal release kinetics of these neuronal mediators is not yet clear. To compare the kinetics of HMGB1 release with CGRP and substance P, we first generated mice in which sensory neurons can be activated with temporal and spatial precision using an external source of blue light. Mice expressing Cre recombinase driven by the vesicular glutamate transporter type 2 (VGlut2) promoter were bred with ChR2-eYFP mice, containing a channel rhodopsin (ChR2)-eYFP fusion sequence in the *ROSA26* locus downstream of a loxP-flanked STOP cassette ((Madisen et al. 2012) resulting in Vglut2-Cre/ChR2-eYFP mice (Yang et al. 2021a). Vglut2 is expressed in peripheral glutamatergic sensory neurons (Scherrer et al. 2010) enabling the optogenetic stimulation of sensory neurons in the paw. Cultured sensory neurons were harvested from DRG of Vglut2-Cre/ChR2-eYFP mice and stimulated in vitro using 470 nm light. Consistent with previous study (Yang et al. 2021a), a time-dependent increase in the extracellular HMGB1 concentrations is observed following optogenetic stimulation of sensory neurons with a delay, increasing significantly only after 60 min (Supplementary Fig. 1A). In contrast, calcitonin gene-related peptide (CGRP) is released as early as 10 min post-stimulation, followed in time by substance P (Supplementary Fig. 1B-C). As all three mediators were significantly elevated at 60 min following stimulation, this time was selected for further in vitro experiments.

**Fig. 1:**
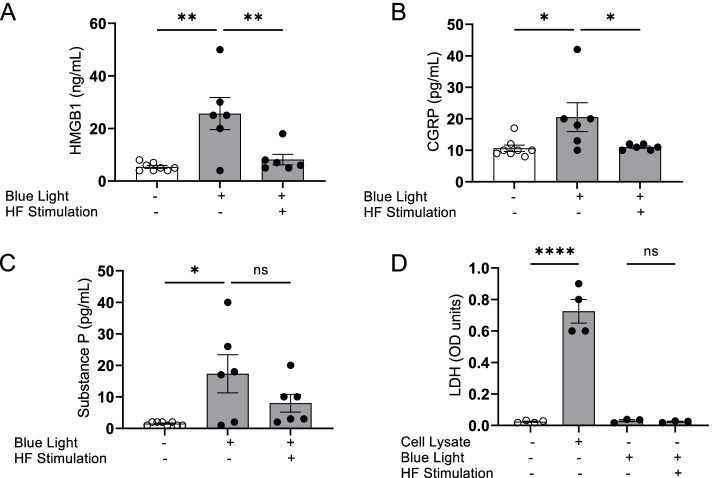
HFES attenuates neuroinflammatory mediators released by optogenetic sensory neuron activation. **A**-**D** Dorsal root ganglia (DRG) sensory neurons harvested from Vglut2-Cre/ChR2-eYFP mice were cultured on multi-electrode arrays (MEA) culture plates for 48–72 h. Cells were simultaneously stimulated with 470 nm (Blue) LED light and HFES for 15 min. Sixty minutes post-stimulation, cell supernatants were harvested and **A** HMGB1, **B** CGRP and **C** substance P measured. Data are represented as individual experimental data point with mean ± SEM. *N* = 6-8 per group. One-way ANOVA followed by Tukey’s multiple comparisons test between groups. *p < 0.05., **p < 0.01, ns: not significant. **D** Neither optogenetic stimulation or HFES induces neuronal cell death. Cell viability was measured by LDH release with cell lysate included as positive control. *n* = 3-4 separate experiments, and each performed in duplicate. Data are represented as individual experimental data point with mean ± SEM. One-way ANOVA followed by Tukey’s multiple comparisons test between groups: ****p < 0.0001. ns: not significant

Next, to directly evaluate whether HFES regulates the release of pro-inflammatory molecules by optogenetically-activated sensory neurons, we adapted an in vitro system for directly stimulating the cultured sensory neurons. Sensory neurons harvested from Vglut2-Cre/ChR2-eYFP mice were cultured on multi-electrode array (MEA) plates and subjected to simultaneous optogenetic activation and HFES. After 48 h of culture, sensory neurons were stimulated using 470 nm light, (20 Hz, 10% duty cycle for 30 min). Optogenetic activation results in release of HMGB1, CGRP and substance P levels into the cell supernatants (Fig. 1A-C). Concurrent HFES of cultured sensory neurons using the MEA electrode grid significantly suppressed HMGB1 (HMGB1: Blue light = 25.7 ± 6.1 ng/mL, Blue light + HF Stimulation = 8.2 ± 2.0 ng/mL, ** *P* < 0.01, Fig. 1A) and CGRP (CGRP: Blue light = 20.5 ± 4.6 pg/mL, Blue light + HF Stimulation = 11.0 ± 0.4 pg/mL, *P < 0.05, Fig. 1B) release by cultured sensory neurons. Released substance P also exhibited a non-significant decrease following high-frequency stimulation (Substance P: Blue light = 17.3 ± 6 pg/mL, Blue light + HF Stimulation = 8.0 ± 2.8 pg/mL, Fig. 1C). Optogenetic activation or high-frequency stimulation do not cause cell death in cultured sensory neurons as documented by the absence of LDH in the media, a soluble cytoplasmic enzyme released upon membrane disruption following cell death (Fig. 1D). Collectively, these data show that HFES reduces HMGB1 and neuroinflammatory peptide release by activated sensory neurons without causing cell death.

### HFES inhibits capsaicin- induced HMGB1, CGRP and substance P release

**Fig. 2:**
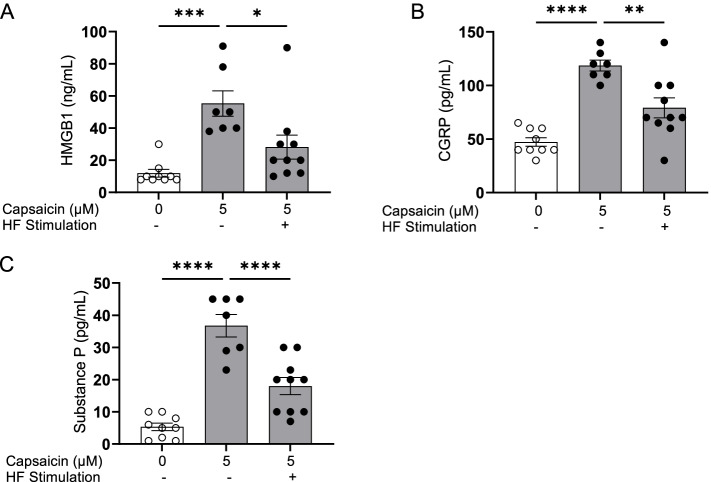
HFES attenuates capsaicin- induced HMGB1, CGRP and substance P release. DRGs from C57BL/6 mice were harvested and plated on MEA plates for 48–72 h. Cells were simultaneously stimulated with capsaicin (5 μM) and high frequency electrical stimulation for 15 min. Sixty minutes post-stimulation, cell supernatants were harvested and **A** HMGB1, **B** CGRP and **C** substance P measured. Data is represented as individual experimental data points with mean ± SEM. *N* = 6-8 per group. One-way ANOVA followed by Tukey’s multiple comparisons test between groups. *P < 0.05, **p < 0.01, ***p < 0.001, ****p < 0.0001

Previous studies have demonstrated that optogenetic activation of TRPV1-expressing sensory neurons induces local inflammation (Cohen et al. 2019). Therefore, we determined whether HFES reduces the release by activated TRPV1- expressing neurons. DRG sensory neurons harvested from C57BL/6 mice were cultured on MEA plates. Cells were concurrently activated by exposure to the TRPV1 agonist capsaicin and subjected to simultaneous HFES. Exposure to capsaicin induces significant increases in levels of HMGB1, CGRP and substance P in the supernatant (Fig. 2A-C). Application of HFES significantly attenuates capsaicin-induced increases in HMGB1 (HMGB1: control = 11.9 ± 2.4 ng/mL, capsaicin = 55.3 ± 7.9 ng/mL, capsaicin + HF stimulation = 28.2 ± 7.4 ng/mL, *P < 0.05, Fig. 2A), CGRP (CGRP: control = 47.2 ± 4.0 pg/mL, capsaicin = 118.6 ± 5.1 pg/mL, capsaicin + HF stimulation = 79.1 ± 9.3 pg/mL, **P < 0.01, Fig. 2B) and substance P (Substance P: control = 5.3 ± 1.2 pg/mL, capsaicin = 36.7 ± 3.5 pg/mL, capsaicin + HF stimulation = 18.0 ± 2.7 pg/mL, ****P < 0.0001, Fig. 2C). These results confirm that HFES attenuates activated sensory neuron release of neuroinflammatory mediators.

### HFES attenuates neuroinflammation and pain behavior induced by optogenetic activation of sensory neurons

**Fig. 3:**
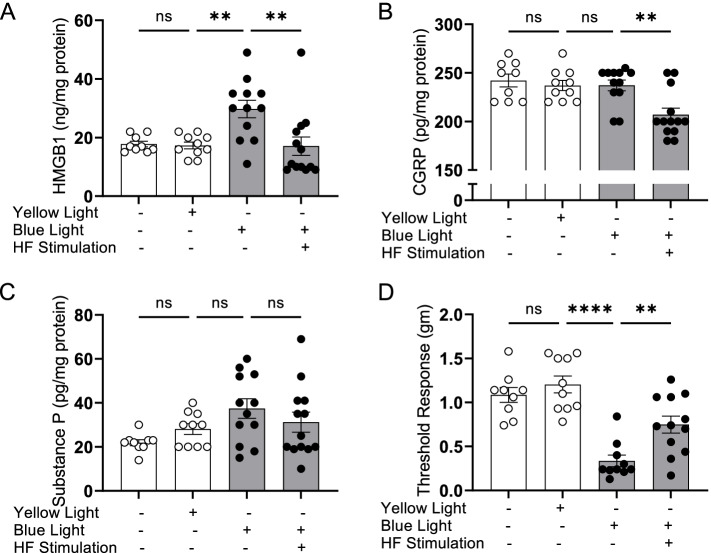
HFES reduces optogenetically-induced HMGB1 release and mechanical allodynia. Vglut2-Cre/ChR2-eYFP mice were anesthetized and subjected to optogenetic stimulation using 470 nm LED (blue) or 595 nm LED (yellow light) for 15 min on the dorsum of the right hind paw, followed by transcutaneous HFES of the sciatic nerve for 10 min (5 min perpendicular and parallel to the sciatic nerve, VGlut2-ChR2-YFP mice only (**A**-**C**). Levels of HMGB1, CGRP and substance P were measured in paw interstitial fluid at 5 h post-stimulation. Data is represented as individual mouse data points with mean ± SEM. One-way ANOVA followed by Tukey’s multiple comparisons test between groups. *N* = 5-12 per group. *: *P* < 0.05. **: *P* < 0.01. ns: not significant. **D** Mechanical hypersensitivity was assessed 5 h later using von Frey filaments. HFES significantly improves optogenetically-induced mechanical hypersensitivity as compared to sham stimulated mice. Data is represented as individual mouse data points with mean ± SEM. One-way ANOVA followed by Tukey’s multiple comparisons test between groups. *N* = 5-12 per group. **P < 0.01. **** *P* < 0.0001. ns: not significant

To determine whether HFES is sufficient to modulate the acute inflammation and pain behavior induced by optogenetic stimulation of sensory neurons in vivo, we stimulated the dorsal paw in Vglut2-Cre/ChR2-eYFP mice using 470 nm light (3 Hz, 20% DC, 4.7 mW, 15 min; Supplementary Fig. 2A-D). As expected, exposure to blue light did not alter the levels of inflammatory mediators or mechanical hypersensitivity in the stimulated paws of wild type (C57BL/6) mice which do not have light-sensitive channel-rhodopsin expressed by sensory neurons (Supplementary Fig. 3A-D). In contrast, a single session of HFES attenuates HMGB1 release in the paw induced light stimulation in Vglut2-Cre/ChR2-eYFP mice (Fig. 3A; HMGB1: yellow light = 17.3 ± 1.2 ng/mg total protein, blue light = 29.8 ± 3.0, blue light + HF stimulation = 17.1 ± 3.1* ng/mg protein, *N* = 9-12 mice per group, *P < 0.05). Although CGRP and substance P are both implicated in the pathogenesis of neuroinflammatory pain, no significant changes were observed in tissue levels of substance P after optogenetic activation or HFES (Fig. 3B-C). In agreement with the elevated levels of neuroinflammatory mediators, optogenetic stimulation on the paw also induced mechanical allodynia which was significantly reduced by concomitant HFES (Fig. 3D). These results suggest that the beneficial effects of HFES depend directly upon the modulation of tissue levels of HMGB1 and less so on CGRP and substance P.

### HFES reduces neuroinflammatory mediators and mechanical hypersensitivity associated with chronic nerve injury

**Fig. 4:**
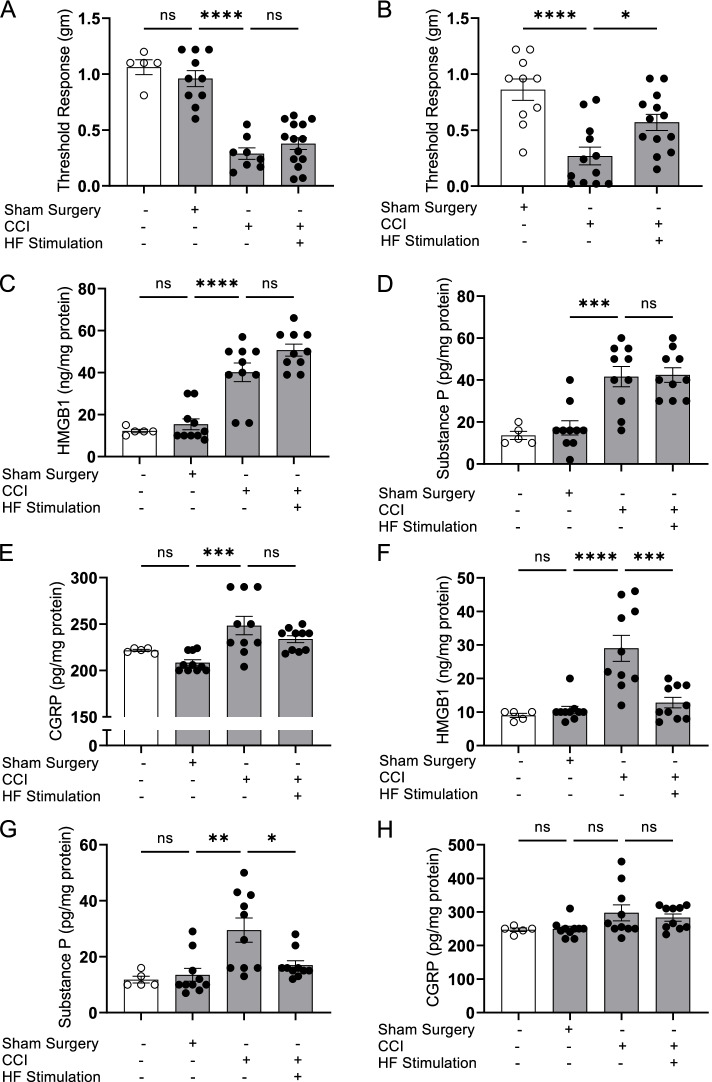
HFES ameliorates chronic constriction injury-induced hyperalgesia and HMGB1 release in mice. Wild type C57BL/6 mice were subjected to chronic constriction injury (CCI) of the right sciatic nerve or sham surgery. Two weeks after sciatic nerve ligation or sham surgery, animals were anesthetized, and transcutaneous HFES was applied for 5 min perpendicularly and parallel to the injury site once a day **A** for 1 day or **B** for 3 consecutive days. Mechanical hypersensitivity was assessed at 24 h after the last stimulation. Data is represented as individual mouse data points with mean ± SEM. One-way ANOVA was used followed by Tukey’s multiple comparisons test between groups. *N* = 5-14 per group. *P < 0.05, ****P < 0.0001. ns: not significant. **C**-**E** CCI animals subjected to transcutaneous HFES once a day for 1 day were euthanized post-pain assessment and levels of **C** HMGB1, **D** substance P, and **E** CGRP in interstitial fluid of the inflamed paws were measured. Data is represented as individual mouse data points with mean ± SEM. One-way ANOVA was used followed by Tukey’s multiple comparisons test between groups. *N* = 5 for normal, *N* = 10 for the other groups. ***P < 0.001, ****P < 0.0001. ns: not significant. **F**-**H** CCI animals stimulated once a day for 3 consecutive days were euthanized post-mechanical hypersensitivity assessment and levels of **F** HMGB1, **G** substance P, and **H** CGRP were measured in the interstitial fluid of the inflamed paws. Data is represented as individual mouse data points with mean ± SEM. One-way ANOVA followed by Tukey’s multiple comparisons test between groups. *N* = 5 for normal, *N* = 10 for the other groups. *: *P* < 0.05, **P < 0.01, ***P < 0.001, ****P < 0.0001. ns: not significant

**Fig. 5:**
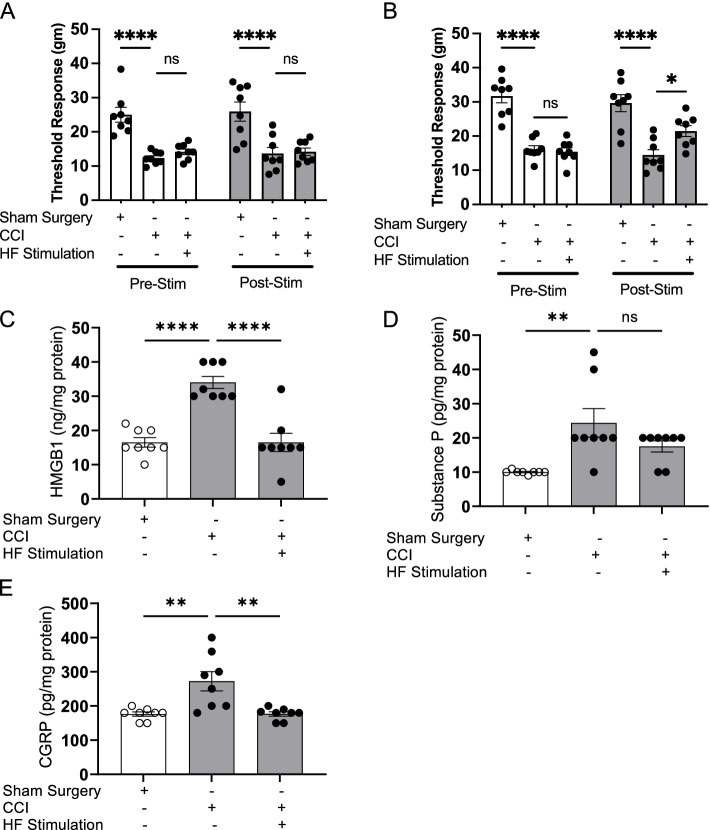
HFES ameliorates chronic constriction injury-induced hyperalgesia and HMGB1 release in rats. Male Sprague-Dawley rats were subjected to CCI of the right sciatic nerve or sham surgery. Two weeks after sciatic nerve ligation surgery or sham surgery, animals were anesthetized, and transcutaneous high frequency electrical stimulation was applied for 5 min perpendicularly and parallel to the injury site, for a total of 10 min once a day **A** for 1 day or **B** for 3 consecutive days. Mechanical hypersensitivity (von Frey) was assessed at 24 h after the last stimulation. Data are represented as individual rat data points with mean ± SEM. Two-way ANOVA was used followed by Tukey’s multiple comparisons test between groups. *N* = 8-12 per group. *P < 0.05, ****P < 0.0001. ns: not significant. **C**-**E** CCI animals stimulated once a day for 3 consecutive days were euthanized post-mechanical hypersensitivity assessment and levels of **C** HMGB1, **D** substance P, and **E** CGRP were measured in the interstitial fluid of the inflamed paws. Data are represented as individual rat data points with mean ± SEM. One-way ANOVA was used followed by Tukey’s multiple comparisons test between groups. *N* = 7-8 per group, **P < 0.01, ****P < 0.0001. ns: not significant

We have previously demonstrated that HMGB1 released by sensory neurons following constrictive nerve injury (CCI) causes tissue inflammation and hyperalgesia (Yang et al. 2021a). To determine whether HFES improves hyperalgesia and chronic pain by modulating neuroinflammatory mediator release by nociceptors in vivo, mice were subjected to CCI and allowed to recover for 15 days. Using the TrueRelief® device (20.6 kHz, 5 min perpendicular followed by 5 min parallel at the injury site for a total of 10 min stimulation) once, or once a day for 3 days showed that the more intensive HFES treatment protocol significantly attenuates CCI-induced mechanical hypersensitivity (Fig. 4A-B). To determine the levels of pro-inflammatory mediators released into the inflamed paw, we collected interstitial fluid using the EXPEL method which showed that the more intensive HFES treatment protocol reduces HMGB1 (normal = 9.0 ± 0.6 ng/mg protein, sham surgery = 10.6 ± 1.1 ng/mg protein, CCI = 29.0 ± 3.9****, and CCI + HF stimulation = 12.8 ± 1.6*** ng/mg protein, *N* = 10 per group, ****P < 0.0001 vs. sham group, ***P < 0.001 vs. CCI group, Fig. 4F), but not in animals subjected to a single HFES treatment (Fig. 4C). In response to nerve injury, CGRP and substance P are also released and play a role in inducing inflammatory responses and hyperalgesia (Seybold 2009; Lukacs et al. 2017). An increase in substance P levels (Fig. 4D, G), but not in CGRP levels (Fig. 4H), is observed in paw tissues after sciatic nerve injury, which is significantly reduced when animals are subjected to 3 days of HFES (Fig. 4G). Next, using the same protocol we evaluated the therapeutic potential of HFES in modulating mechanical hyperalgesia in rats undergoing CCI. Like mice, rats subjected to CCI exhibit mechanical allodynia (Fig. 5A, B) which HFES reduces. The increased levels of HMGB1 and CGRP in the inflamed paw tissues of rats subjected to CCI of the sciatic nerve are reduced significantly following 3 days of HFES, whereas no significant changes are observed in substance P levels (HMGB1: sham surgery = 16.5 ± 1.4 ng/mg protein, CCI = 34.0 ± 1.8 ng/mg protein, CCI + HF stimulation = 16.5 ± 2.7 ****ng/mg protein, ****P < 0.0001 vs. CCI group, *N* = 8 per group. Figure 5C; CGRP: sham surgery = 176.3 ± 6.3 ng/mg protein, CCI = 272.4 ± 28.1 ng/mg protein, CCI + HF stimulation = 176.6 ± 7.1 **ng/mg protein, **P < 0.01 vs. CCI group, Fig. 5E). In comparison, elevated levels of substance P in the paw tissues were not significantly altered by the HFES (Fig. 5D).

## Discussion

High frequency electrical stimulation has achieved significant clinical success in the treatment of pain and in preclinical models of non-cancer chronic pain, neuropathic pain, and osteoarthritis pain (Kumar et al. 2007; Echeverria-Villalobos et al. 2021; Al-Kaisy et al. 2014; Grabow et al. 2003). However, the underlying mechanism of action is unexplained and patients experience a wide range of outcomes due to unknown variables which hamper the efficacy of treatment. The data presented here show that HFES targeting sensory nerves inhibits the release of key molecular triggers of neuroinflammation both in vitro and in vivo. As inflammation is inherently a self-amplifying process which drives ongoing pain and tissue injury, this perspective offers an etiological explanation for HFES efficacy. Current clinical and proposed disease modifying treatments of the neuroinflammatory state have been directed towards neutralization of the signaling and effects of specific pro-inflammatory components of a complex inflammatory cascade, e.g., targeting CGRP (De Matteis et al. 2020), substance P (Johnson et al. 2017), or HMGB1 (Yang et al. 2021b; Andersson et al. 2018). As HFES inhibits the release of multiple inflammatory mediators which have differing downstream pathophysiologic effects, this approach offers the potential for a therapy directed earlier in a preventative manner in the disease process. Additionally, demonstrated efficacy of HFES in the CCI model of nerve injury after several weeks shows that this approach is also effective as a treatment for established inflammation and pain.

The neurophysiological and molecular mechanisms by which HFES inhibits nociceptor release of proinflammatory molecules remains to be explored by future study. Spinal cord stimulation (SCS) has been used clinically since the 1960s in the form of applied low frequency energy which produced associated paresthesia. This suggested “gate control” theory (Melzack and Wall 1965), which postulates that non-nociceptive signals (paresthesia) inhibits nociceptor signal transmission. However, modern SCS devices now use high frequency stimulation (Sayed et al. 2020), which elicits no associated paresthesia, invalidating the gate control hypothesis (Echeverria-Villalobos et al. 2021). Other theories contend that electrical stimulation suppresses electrical signaling necessary to carry pain signals either by the direct blocking of nerve conduction (Reboul and Rosenblueth 1939; Rosenblueth and Reboul 1939), or by the downstream inhibition of neurons proximal to the injury site (Arle et al. 2016). An expanded explanation theorizes that differential blocking occurs, in which stimulation blocks paresthesia-producing large fibers, while activating other fibers which do not contribute to paresthesia, that in turn sufficiently inhibits downstream signaling of pain by wide dynamic range neurons occurs (Arle et al. 2016). Other theories which pertain to transcutaneous stimulation postulate that activation of endogenous opioid pathways (Claydon et al. 2011) and reduction of proinflammatory cytokines (Gürgen et al. 2013) contribute to efficacy through either central or peripheral actions (Lin et al. 2020). In contrast to these theories, the simplest explanation of the results of the current experiments is that HFES directly blocks sensory nerve secretory activity, and therefore, subsequent neuroinflammatory processes.

The data provided by the CCI model is interesting from the perspective that 15 days after the initiation of injury, neuroinflammatory mediators are present within the local receptive field of nociceptors in the hind paw. In this model, a majority of nociceptors are lost within a few days of injury and only slowly regenerate after a period of weeks, depending on the fiber type. For example, CGRP positive epidermal fibers are reduced by ~ 90% by day 3 following sciatic nerve ligation, returning to normal only at 8 weeks (Peleshok and Ribeiro-da-Silva 2011), which is mirrored by pain-related behaviors (Lindenlaub and Sommer 2002). The variable loss of sciatic nerve fibers innervating the foot, and therefore different pro-inflammatory mediators presumably underlies the variability in the observed concentrations of CGRP and substance P we observed. In contrast, HMBG1 appears to be a more robust inflammatory index which may be explained by its presence within larger, better-preserved innervating or regenerating neurons in this model. Thus, inhibition of HMGB1 release, a more proximal member of the inflammatory cascade (which includes many other downstream pro-inflammatory cytokines not evaluated in the current study) is likely a critical target of HFES.

Although among the many methods evaluating pain behaviors in rodents, mechanical allodynia and hyperalgesia assessed by the application of von Frey filaments of varying forces is one of the most common measurements used in pain behavior in rodents (Drew et al. 2007). One limitation of this study is that we did not use experimental methodologies to measure more “natural” behaviors in animals. These systems (such as home cage testing, conditioned place preference, conditioned place aversion) could enable prolonged and longitudinal recordings and provide large continuous measures of spontaneous non-stimulus-evoked behavior that can be analyzed across multiple time scales. These tests will be used for our future studies.

Future work is needed to better define optimal HFES peripheral nerve stimulation protocols which presumably will vary depending on the underlying pathology intended to be treated. These studies will require a more complete evaluation of timing (how long, how frequently), optimum waveform (which depends on the anatomy of surrounding non-neural tissues and fiber size distribution of the intended target nerves), and determination of optimal placement of electrodes with respect to individual sensory nerves. Of particular importance is understanding the onset and duration of effect which is complicated in vivo by the presence and plasticity of higher order neurons. As one illustrative example, another therapeutic approach to treatment of the neuroinflammation and pain elicited in the CCI model is pulsed radio frequency applied to the sciatic nerve at the site of injury. Here, a single treatment results in delayed efficacy as assessed by changes in thermal and mechanical threshold changes in the hind paw, such that progressive improvements are noted over the course of 14 days (Ren et al. 2018). Whether HFES shows similar effects in this model remains to be determined.

## Conclusion

HFES modulates neuronal HMGB1-mediated neuroinflammation and neuropathic pain. Insight into the mechanistic effects of HFES will be impactful as HFES is currently used rather empirically as a treatment strategy for a number of pain conditions, and knowing the targeted effects on neuronal HMGB1 will become imperative to design future patient-oriented therapeutic paradigms.

## Supplementary Information

**Additional file 1: Supplementary Figure 1.** Light activated sensory neurons release inflammatory mediators. DRG sensory neurons harvested from Vglut2-Cre/ChR2-eYFP mice were cultured for 48–72 h and then stimulated with blue light (470 nm) at 20 Hz, 10% duty cycle for 15 min. Supernatant was harvested at indicated time points, and levels of HMGB1 (A), substance P (B) and CGRP (C) were quantified. *N* = 4-5 per group. *P < 0.05, **P < 0.01, ***P < 0.001.

**Additional file 2: Supplementary Figure 2.** Experimental setup for optogenetic and HFES. (A) Animals were induced and maintained under anesthesia using isoflurane (1.5-2%). The right hind paw was extended and secured to minimize movement during stimulation. (B) The LED (470 or 595 nm) was positioned 1 cm above the right hind paw. (C) Optogenetic stimulation was applied to the right hind paw for 15 min (3 Hz, 20% Duty Cycle). (D) Immediately after optogenetic stimulation, two probe tips covered with a conductive gel (Spectra 360 gel) were positioned above the sciatic nerve. Transcutaneous HFES (20.6 kHz) was applied for 5 min parallel and perpendicular to the sciatic nerve for a total of 10 min. After electrical stimulation, the animal was allowed to recover in a clean cage.

**Additional file 3: Supplementary Figure 3.** Acute optogenetic stimulation does not induce hyperalgesia or the release of inflammatory mediators in wild type mice or primary sensory neurons. Wild type (C57BL/6) mice were anesthetized and subjected to optogenetic stimulation using 470 nm LED (blue) or 595 nm LED (yellow light) for 15 min on the dorsum of the right hind paw. (A) Mechanical hypersensitivity was assessed 5 h later, using von Frey filaments. Blue or yellow light stimulation did not induce any mechanical hypersensitivity to wild type animals. Data is represented as individual mouse data points with mean ± SEM. One-way ANOVA followed by Tukey’s multiple comparisons test between groups. *N* = 5 per group. ns: not significant. (B-D) Levels of HMGB1, CGRP and substance P were measured in the interstitial fluids of the paws at 5 h post-stimulation. Data is represented as individual mouse data points with mean ± SEM. One-way ANOVA followed by Tukey’s multiple comparisons test between groups. *N* = 5-10 per group. ns: not significant.

## Data Availability

The datasets during and/or analyzed during the current study available from the corresponding authors on reasonable request.

## Abbreviations

HFES: High-frequency electrical nerve stimulation
HF: High frequency
HMGB1: High mobility group box 1
CGRP: Calcitonin gene-related peptide
SP: Substance P
LDH: Lactate dehydrogenase
VGlut-2: Vesicular glutamate transporter type-2
ChR2: Channelrhodopsin-2
YFP: Yellow fluorescent protein
TrpV1: Transient receptor potential cation channel subfamily V member 1
DRG: Dorsal root ganglia
CCI: Chronic constriction injury
SCS: Spinal cord stimulation
HBSS: Hank’s balanced salt solution
PBS: Phosphate buffered saline
MEA: Multi-electrode array
PEDOT-CNT: Poly(3,4-ethylenedioxythiophene)-carbon nanotube
ELISA: Enzyme-linked immunosorbent assay
EIA: Enzyme immunoassay
SEM: Standard error of the mean
ANOVA: Analysis of variance
KO: Knockout
WT: Wild type
