# Supplementary figures and images for: High-frequency electrical stimulation attenuates neuronal release of inflammatory mediators and ameliorates neuropathic pain
Source: Bioelectron Med. 2022 Oct 5;8:16. doi: 10.1186/s42234-022-00098-8 (PMC9533511; doi:10.1186/s42234-022-00098-8)

# Supplementary Figure 1

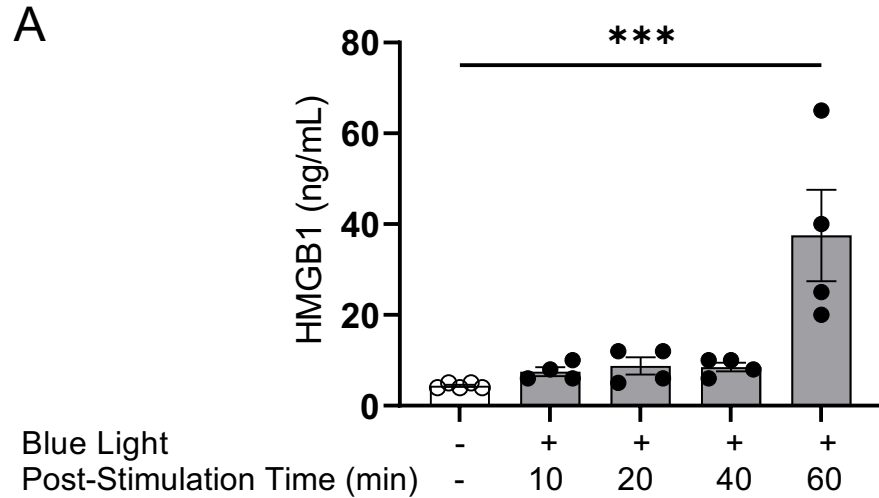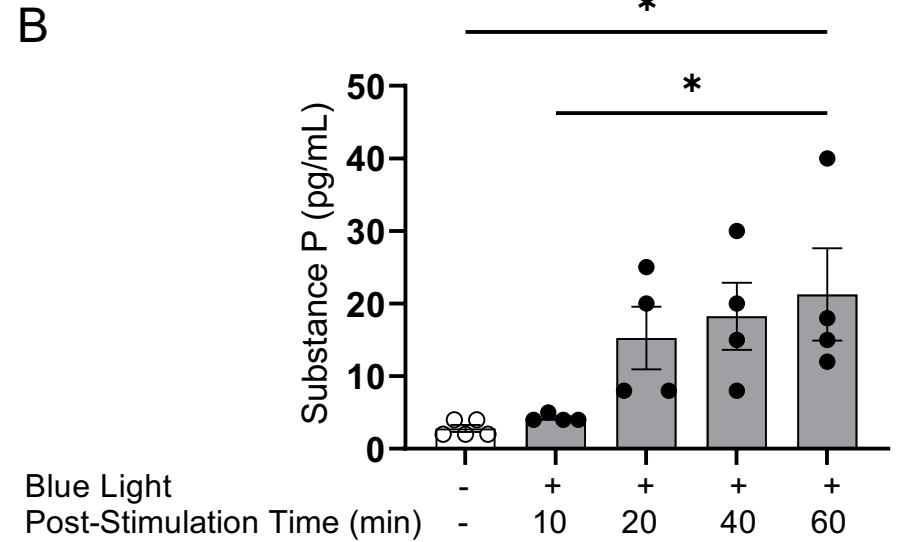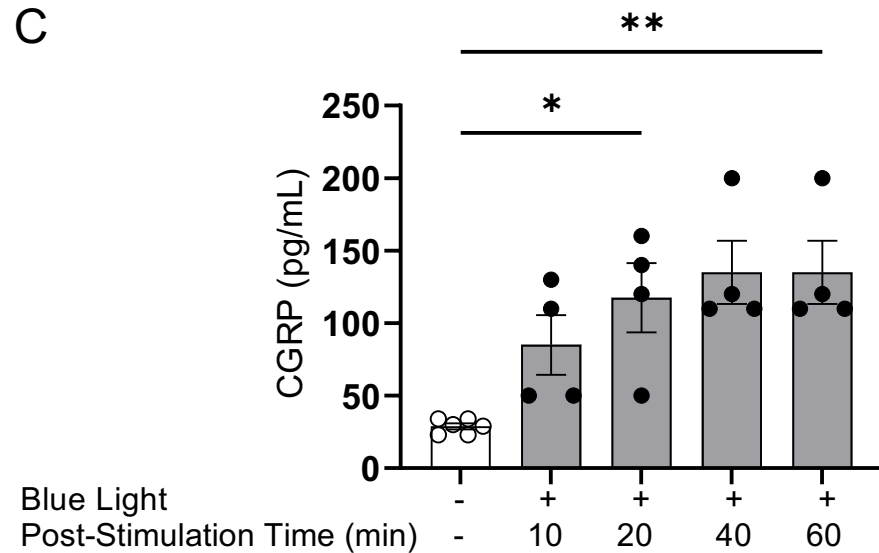

Supplement: Supplementary file 1 — Additional file 1: Supplementary Figure 1. Light activated sensory neurons release inflammatory mediators. DRG sensory neurons harvested from Vglut2-Cre/ChR2-eYFP mice were cultured for 48–72 h and then stimulated with blue light (470 nm) at 20 Hz, 10% duty cycle for 15 min. Supernatant was harvested at indicated time points, and levels of HMGB1 (A), substance P (B) and CGRP (C) were quantified. N = 4-5 per group. *P < 0.05, **P < 0.01, ***P < 0.001. [file 42234_2022_98_MOESM1_ESM.pdf]

# Supplementary Figure 2

A

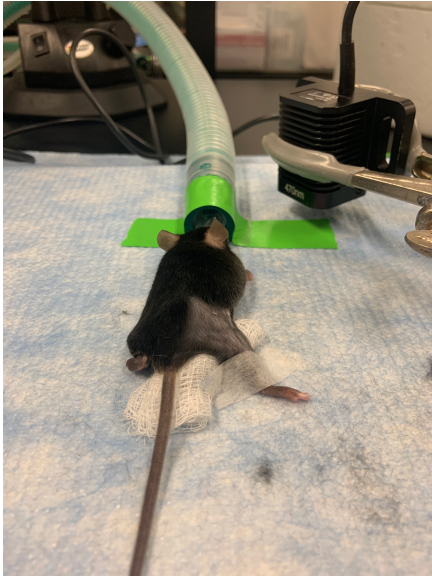

B

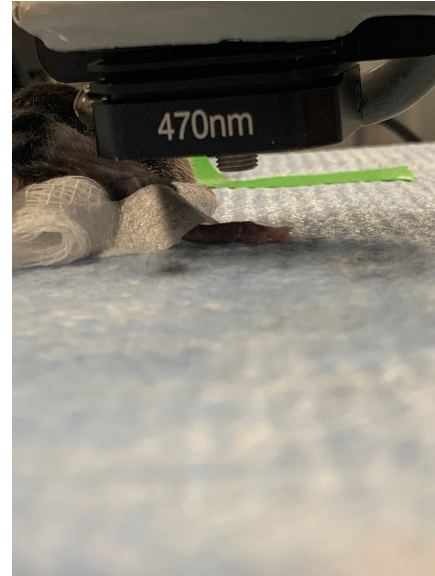

C

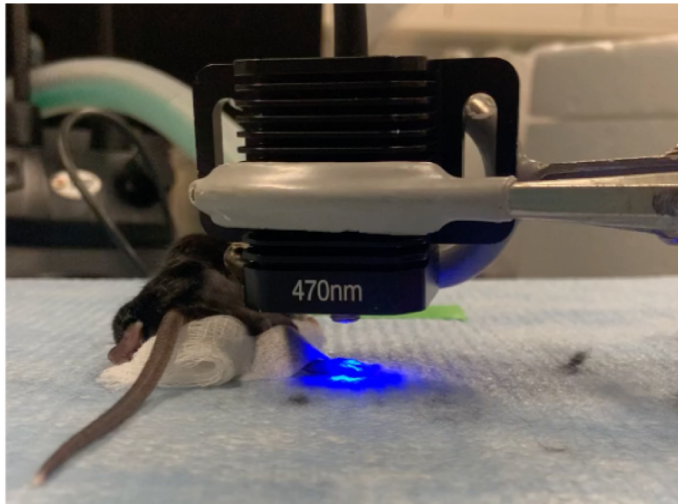

D

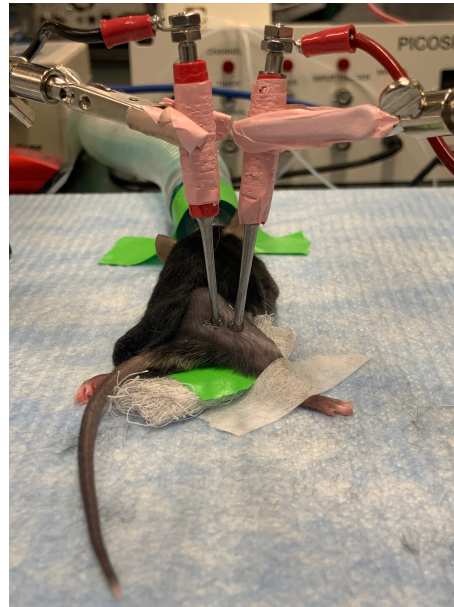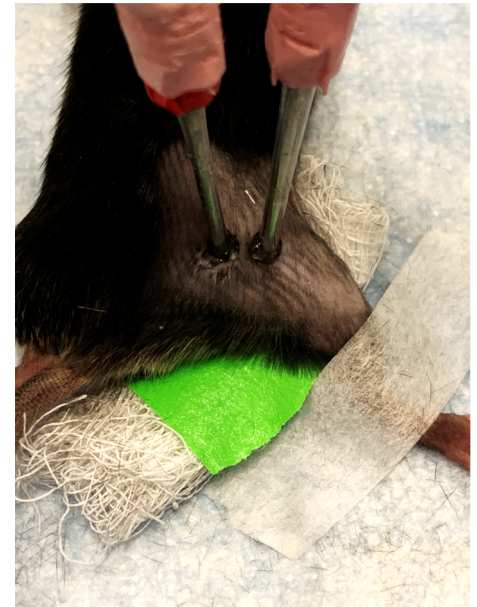

Supplement: Supplementary file 2 — Additional file 2: Supplementary Figure 2. Experimental setup for optogenetic and HFES. (A) Animals were induced and maintained under anesthesia using isoflurane (1.5-2%). The right hind paw was extended and secured to minimize movement during stimulation. (B) The LED (470 or 595 nm) was positioned 1 cm above the right hind paw. (C) Optogenetic stimulation was applied to the right hind paw for 15 min (3 Hz, 20% Duty Cycle). (D) Immediately after optogenetic stimulation, two probe tips covered with a conductive gel (Spectra 360 gel) were positioned above the sciatic nerve. Transcutaneous HFES (20.6 kHz) was applied for 5 min parallel and perpendicular to the sciatic nerve for a total of 10 min. After electrical stimulation, the animal was allowed to recover in a clean cage. [file 42234_2022_98_MOESM2_ESM.pdf]

# Supplementary Figure 3

A

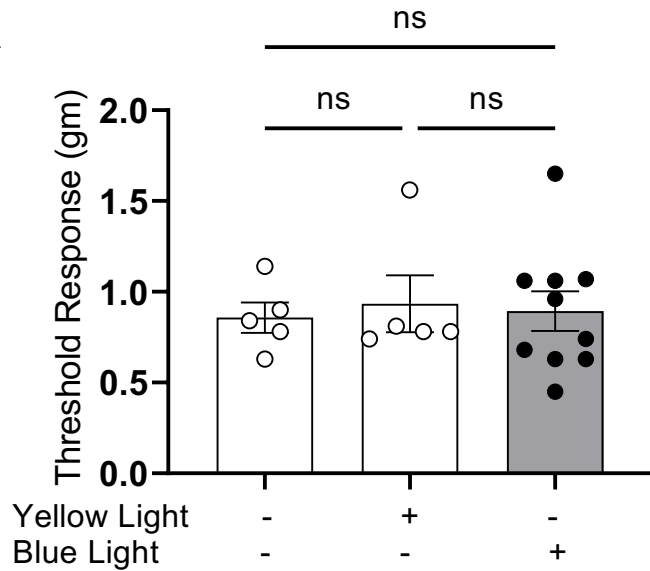

B

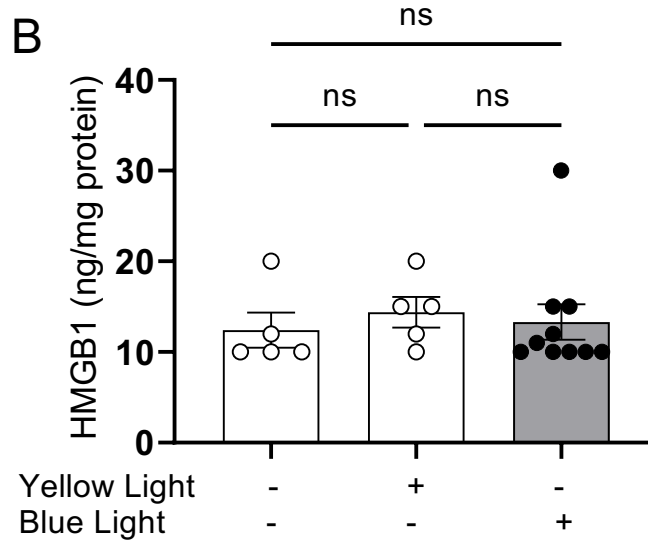

C

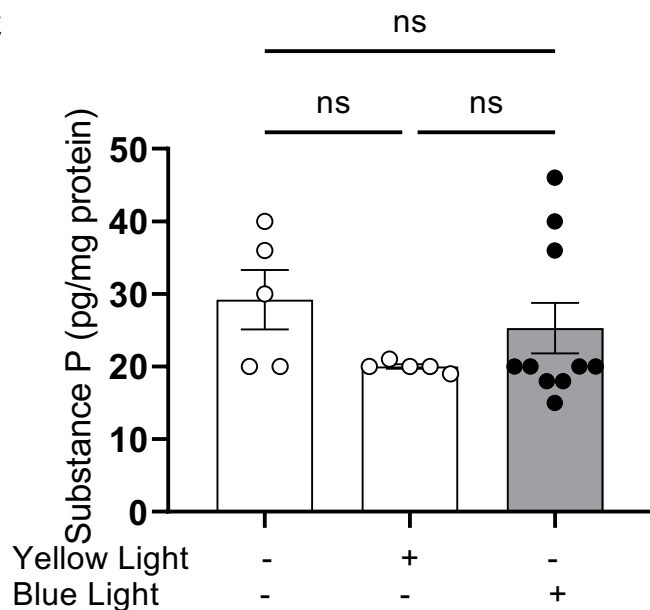

D

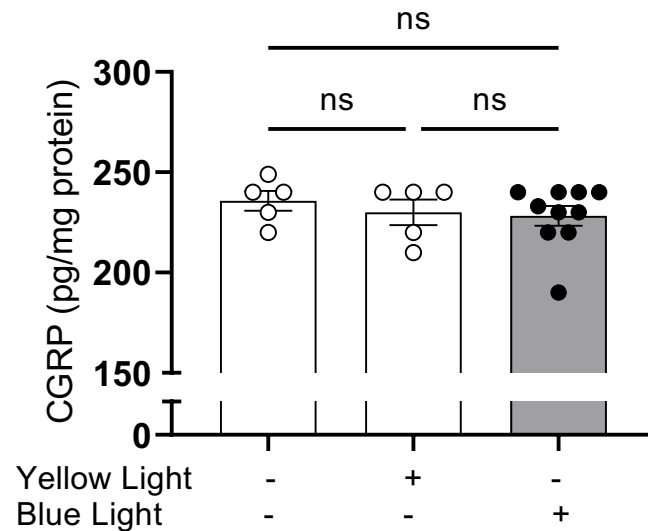

Supplement: Supplementary file 3 — Additional file 3: Supplementary Figure 3. Acute optogenetic stimulation does not induce hyperalgesia or the release of inflammatory mediators in wild type mice or primary sensory neurons. Wild type (C57BL/6) mice were anesthetized and subjected to optogenetic stimulation using 470 nm LED (blue) or 595 nm LED (yellow light) for 15 min on the dorsum of the right hind paw. (A) Mechanical hypersensitivity was assessed 5 h later, using von Frey filaments. Blue or yellow light stimulation did not induce any mechanical hypersensitivity to wild type animals. Data is represented as individual mouse data points with mean ± SEM. One-way ANOVA followed by Tukey’s multiple comparisons test between groups. N = 5 per group. ns: not significant. (B-D) Levels of HMGB1, CGRP and substance P were measured in the interstitial fluids of the paws at 5 h post-stimulation. Data is represented as individual mouse data points with mean ± SEM. One-way ANOVA followed by Tukey’s multiple comparisons test between groups. N = 5-10 per group. ns: not significant. [file 42234_2022_98_MOESM3_ESM.pdf]
